# Supplementary material for: DNA Demethylation Induces Tree Peony Flowering with a Low Deformity Rate Compared to Gibberellin by Inducing PsFT Expression under Forcing Culture Conditions
Source: Int J Mol Sci. 2022 Jun 14;23(12):6632. doi: 10.3390/ijms23126632 (PMC9223562; doi:10.3390/ijms23126632)
Supplement: Supplementary file 1 [file ijms-23-06632-s001.zip › ijms-1739625-supplementary.pdf]

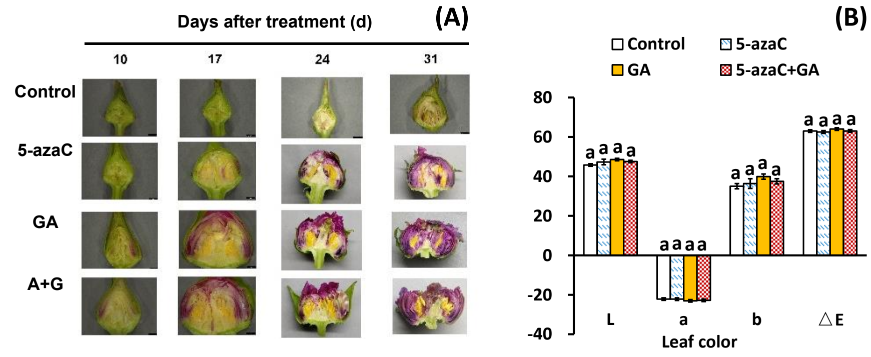


**Figure S1.** Morphological observation by longitudinal cut of buds (A) and statistical analysis of CIE-LAB color in leaves (B) in the four different treatment groups.

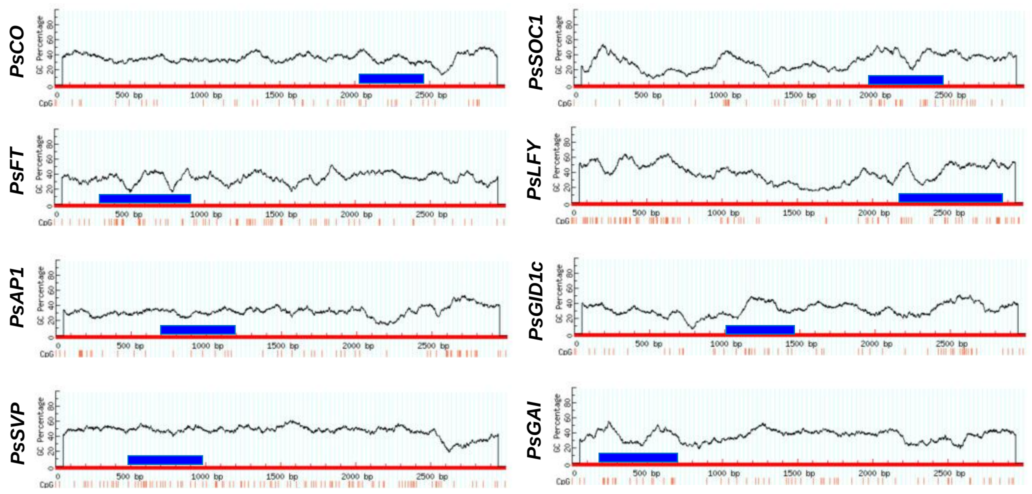


**Figure S2.** The position of CG contents (blue lines) in the promoters of flowering-pathway genes used for DNA methylation determination by McrBC-PCR. The graph is based on MethPrimer (http://www.urogene.org/methprimer). A 3 kb of promoter region upon ATG code of each gene was used for analysis and CpG (red vertical short lines) represents the predication of CG-rich sites.

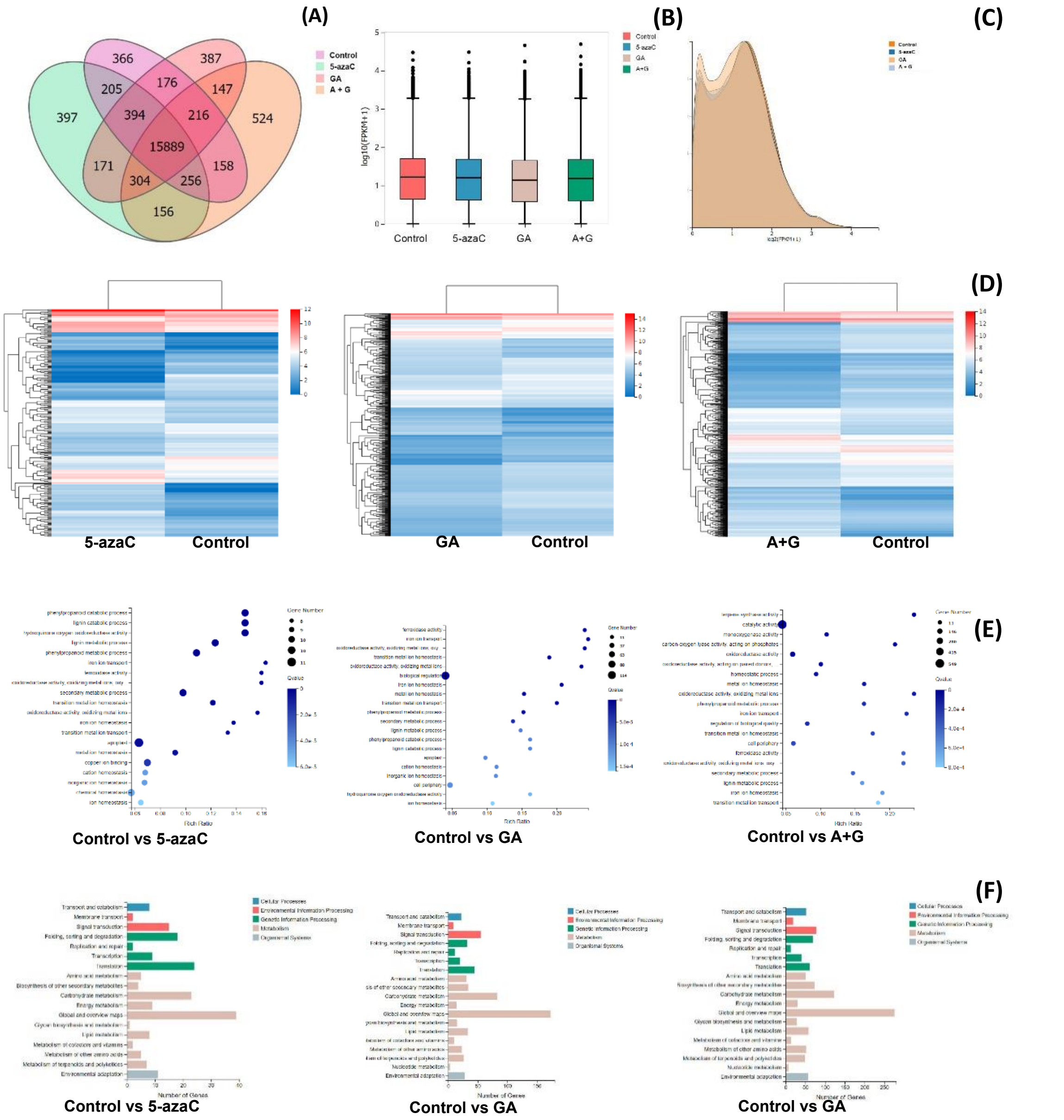


**Figure S3.** Bioinformatics analysis of transcriptome data. (A) Venn diagram of gene number and their distribution in different groups. (B) Box plot comparison of gene expression. (C) Density map of gene expression. X- and Y-axis is $\log_2$ FPKM+1 and gene density, respectively. (D) Cluster heat map of differential gene expression. The horizontal axis represents $\log_2$ FPKM+1 of the sample, and the vertical axis represents genes. (E) Bubble map of DEGs GO enrichment. X-axis represents enrichment ratio, equal to term candidate gene number / term gene number, and Y-axis represents GO Term. The bubble size represents the number of differential genes annotated to a certain GO

Term and the color represents enriched Qvalue. (F) KEGG pathway classification of DEGs. The X-axis represents the number of genes annotated to a KEGG pathway class, and the Y-axis represents the KEGG pathway class.

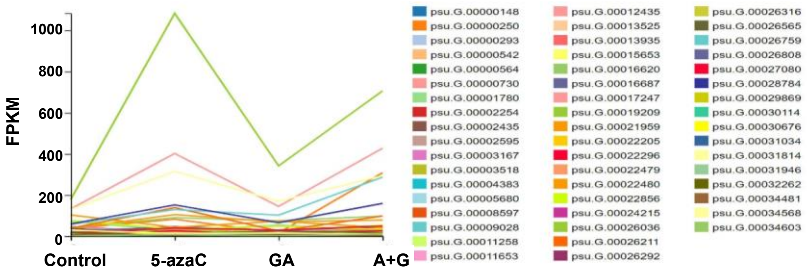


**Figure S4.** Expression pattern of methylation-closer DEGs in four different treatment groups.

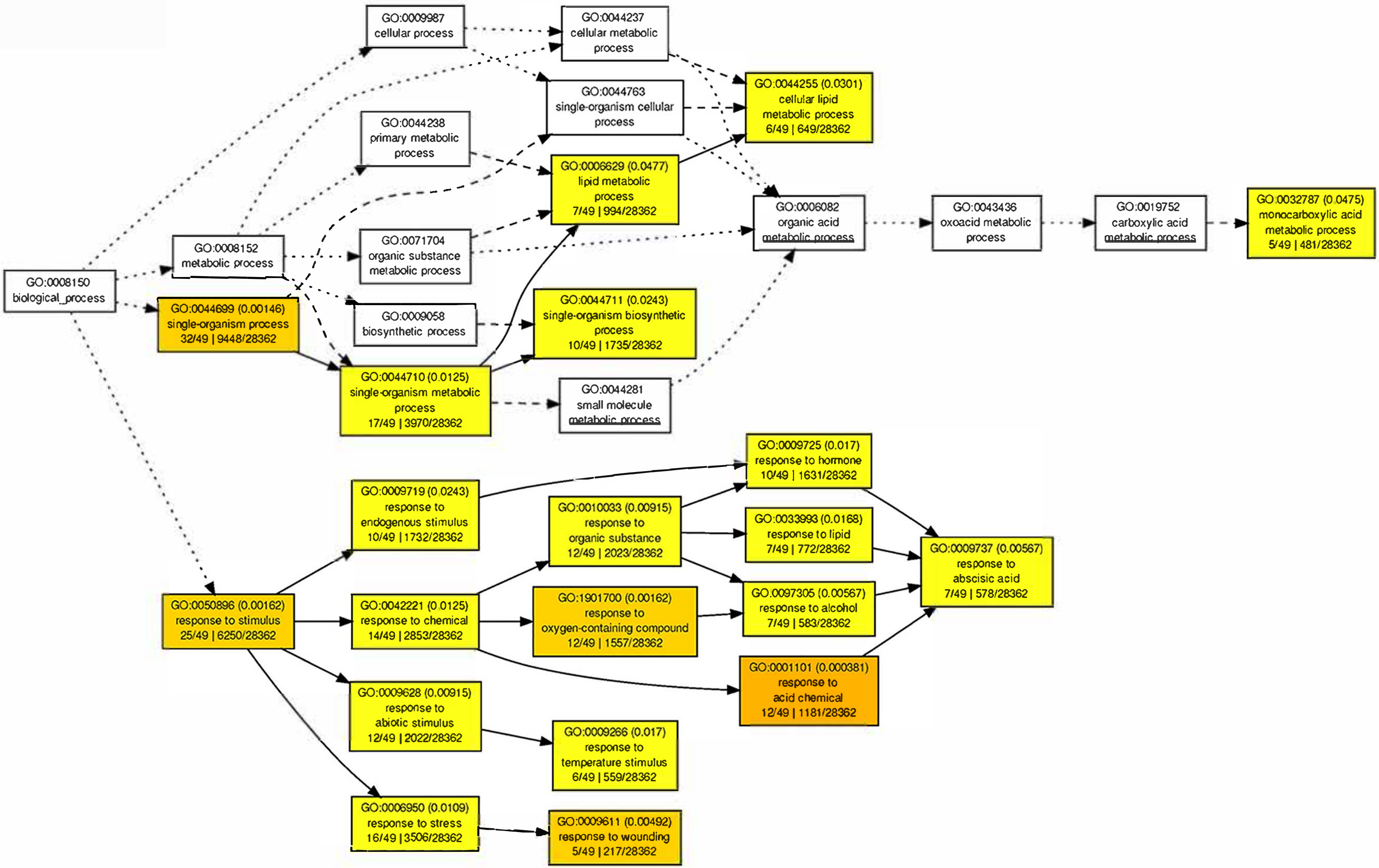


**Figure S5.** GO enrichment path tree of 52 methylation-closer DEGs in biological process compared to Arabidopsis.

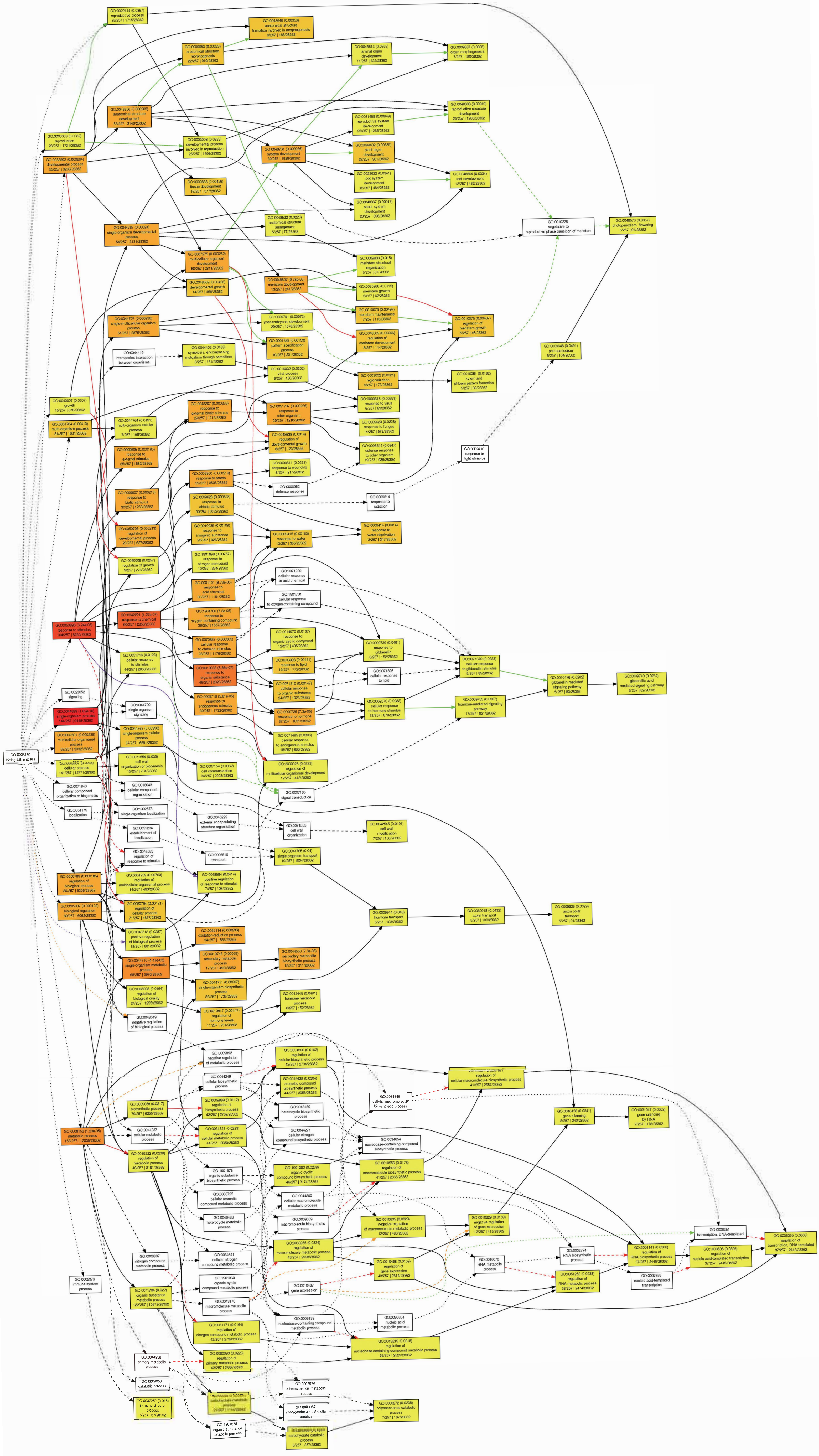

**Figure S6.** GO enrichment path tree of 302 GA-closer DEGs in biological process compared to Arabidopsis.

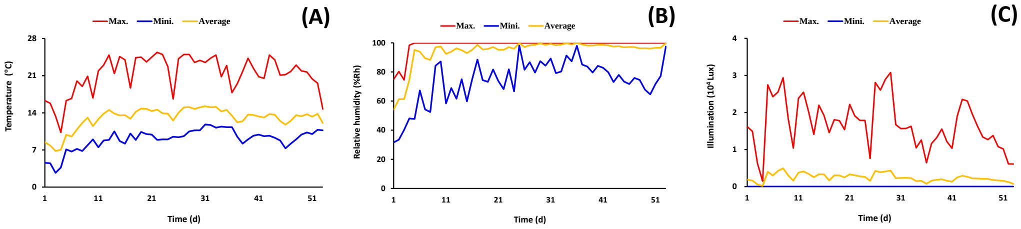


**Figure S7.** The environment conditions in greenhouse during the experiment. (A) Temperature; (B) Relative humidity; (C) Illumination.

**Table S1.** List and information of 52 methylation-closer DEGs

| Gene ID | Related ID in Arabidopsis | FPKM | | | |
|---|---|---|---|---|---|
| | | Control | 5-azaC | GA | A+G |
| psu.G.00000148 | AT2G26690 | 2.81 | 7.75 | 6.67 | 9.58 |
| psu.G.00000250 | AT5G14780 | 36.78 | 82.54 | 16.28 | 308.72 |
| psu.G.00000293 | AT5G23960 | 15.45 | 6.54 | 8.29 | 5.06 |
| psu.G.00000542 | AT5G22390 | 2.33 | 11.6 | 7.42 | 46.01 |
| psu.G.00000564 | AT3G18660 | 2.64 | 7.76 | 6.18 | 6.81 |
| psu.G.00000730 | AT3G56290 | 2.98 | 24.37 | 12.75 | 35.67 |
| psu.G.00001780 | AT1G17840 | 72.41 | 26.87 | 54.98 | 27.18 |
| psu.G.00002254 | AT1G76860 | 0.48 | 7.77 | 3.95 | 8.94 |
| psu.G.00002435 | AT5G03160 | 6.43 | 16.41 | 10.95 | 13.18 |
| psu.G.00002595 | AT5G36110 | 2.26 | 0.16 | 1.4 | 0 |
| psu.G.00003167 | AT2G21630 | 47.27 | 17.99 | 21.43 | 16.79 |
| psu.G.00003518 | AT3G07360 | 17.33 | 41.29 | 18.12 | 36.36 |
| psu.G.00004383 | AT3G45140 | 4.14 | 8.76 | 5.07 | 18.97 |
| psu.G.00005680 | AT2G37170 | 2.76 | 9.82 | 5.81 | 10.24 |
| psu.G.00008597 | AT5G08350 | 0 | 4.75 | 2.43 | 2.87 |
| psu.G.00009028 | AT1G10240 | 0 | 1.62 | 1.01 | 1.74 |
| psu.G.00011258 | AT4G27290 | 2.57 | 0.02 | 2.87 | 0 |
| psu.G.00011653 | No Hit Found | 12.77 | 0 | 15.97 | 0 |
| psu.G.00012435 | No Hit Found | 1.67 | 32.81 | 12.17 | 27.23 |
| psu.G.00013525 | AT1G50460 | 8.27 | 17.13 | 8.16 | 20.08 |
| psu.G.00013935 | AT2G46270 | 6.34 | 21.14 | 16.2 | 29.88 |
| psu.G.00015653 | AT3G22840 | 12.34 | 42.4 | 23.36 | 55.98 |
| psu.G.00016620 | AT4G17460 | 41.81 | 90.18 | 75.46 | 98.21 |
| psu.G.00016687 | AT4G00430 | 22.23 | 46.71 | 27.98 | 44.53 |
| psu.G.00017247 | AT1G26800 | 133.04 | 402.29 | 145.02 | 427.83 |
| psu.G.00019209 | AT1G52690 | 178.73 | 1083.55 | 342.19 | 706.36 |
| psu.G.00021959 | AT5G06570 | 103.12 | 43.28 | 72.77 | 41.99 |
| psu.G.00022205 | AT3G63240 | 9.01 | 1.11 | 8.45 | 0 |
| psu.G.00022296 | AT5G48050 | 10.56 | 26.28 | 12.36 | 26.27 |
| psu.G.00022479 | AT4G27290 | 18.8 | 4.71 | 13.23 | 4.55 |
| psu.G.00022480 | AT4G35350 | 38.25 | 104.92 | 69.57 | 78.29 |
| psu.G.00022856 | AT1G28370 | 76.53 | 35.29 | 48.13 | 33.67 |

| | | | | | |
|---|---|---|---|---|---|
| psu.G.00024215 | AT1G08550 | 19.09 | 0 | 12.37 | 5.54 |
| psu.G.00026036 | AT1G60470 | 2.48 | 36.08 | 5.31 | 17.49 |
| psu.G.00026211 | AT3G24500 | 38.76 | 139.14 | 25 | 98.34 |
| psu.G.00026292 | AT4G03500 | 17.29 | 8.01 | 12.91 | 0 |
| psu.G.00026316 | AT1G21270 | 10.96 | 2.16 | 7.33 | 0 |
| psu.G.00026565 | AT1G20190 | 37.53 | 11.81 | 25.59 | 17.02 |
| psu.G.00026759 | AT5G14740 | 57.32 | 129.38 | 102.59 | 286.84 |
| psu.G.00026808 | AT3G01090 | 0 | 6.74 | 0 | 7.89 |
| psu.G.00027080 | AT4G36830 | 16.16 | 39.29 | 29.3 | 49.83 |
| psu.G.00028784 | AT1G67090 | 59.71 | 152.05 | 65.98 | 159.76 |
| psu.G.00029869 | AT5G57990 | 3.13 | 0 | 2.5 | 0 |
| psu.G.00030114 | AT1G63490 | 4.52 | 0 | 10.98 | 0.19 |
| psu.G.00030676 | AT3G55580 | 4.89 | 16.21 | 6.29 | 12.65 |
| psu.G.00031034 | AT2G26210 | 7.59 | 1.22 | 2.5 | 1.23 |
| psu.G.00031814 | AT5G20620 | 130.97 | 315.76 | 169.84 | 293.93 |
| psu.G.00031946 | AT5G17860 | 12.45 | 4.83 | 10.02 | 4.95 |
| psu.G.00032262 | AT2G38820 | 17.11 | 3.52 | 8.14 | 4.67 |
| psu.G.00034481 | AT4G20140 | 9.07 | 4.45 | 5.11 | 2.54 |
| psu.G.00034568 | AT5G62680 | 28.91 | 14.43 | 15.24 | 9.57 |
| psu.G.00034603 | AT1G78780 | 0.72 | 8.85 | 1.48 | 11.23 |

**Table S2.** List and information of 302 GA-closer DEGs

| Gene ID | Related ID in Arabidopsis | FPKM | | | |
|---|---|---|---|---|---|
| | | Control | 5-azaC | GA | A+G |
| psu.G.00000087 | AT1G67260 | 8.37 | 4.89 | 1.95 | 2.28 |
| psu.G.00000131 | AT1G27660 | 42.63 | 35.58 | 17.74 | 18.13 |
| psu.G.00000266 | AT1G62360 | 7.26 | 15.14 | 1.22 | 0 |
| psu.G.00000272 | AT5G36220 | 20.29 | 19.15 | 6.31 | 9.12 |
| psu.G.00000282 | AT1G74790 | 18.74 | 17.11 | 8.91 | 8.39 |
| psu.G.00000384 | AT4G20140 | 16.23 | 13.29 | 7.36 | 5.13 |
| psu.G.00000754 | AT1G72830 | 37.95 | 23.27 | 8.36 | 8.58 |
| psu.G.00000782 | AT1G10560 | 7.88 | 14.16 | 18.05 | 24.2 |
| psu.G.00000919 | AT5G03680 | 47.47 | 42.82 | 14.66 | 19.64 |
| psu.G.00000921 | AT3G22160 | 143.43 | 75.33 | 38.86 | 44.75 |
| psu.G.00001145 | AT3G45140 | 818.82 | 1101.05 | 2419.71 | 2093.8 |
| psu.G.00001416 | AT2G37130 | 12.3 | 16.52 | 5.22 | 0.98 |
| psu.G.00001427 | AT5G15230 | 566.2 | 539.39 | 269.8 | 125.72 |
| psu.G.00001456 | AT5G06510 | 5.83 | 2.48 | 1.33 | 0.89 |
| psu.G.00001457 | AT1G55790 | 168.99 | 135.28 | 65.64 | 82.26 |
| psu.G.00001668 | AT2G33880 | 37.09 | 20.71 | 9.42 | 7.73 |
| psu.G.00001719 | AT3G50410 | 46.96 | 39.66 | 22.99 | 20.88 |
| psu.G.00001736 | AT4G34240 | 55.99 | 63.86 | 116.67 | 116.56 |
| psu.G.00001953 | AT5G24740 | 11.81 | 9.56 | 5.22 | 5.82 |
| psu.G.00002324 | AT3G54480 | 32.56 | 21.59 | 15.01 | 14.16 |
| psu.G.00002512 | AT5G51560 | 34.97 | 22.34 | 14.03 | 14.47 |
| psu.G.00002587 | AT4G18210 | 8.15 | 16.47 | 31.41 | 32.51 |
| psu.G.00002646 | AT3G52970 | 8.76 | 22.82 | 111.65 | 45.19 |
| psu.G.00002749 | AT5G44010 | 25.29 | 11.11 | 10.1 | 10.99 |
| psu.G.00002758 | AT5G43810 | 249.6 | 197.41 | 121.97 | 107.89 |
| psu.G.00002959 | AT3G12500 | 29.05 | 44.37 | 75.79 | 70.31 |
| psu.G.00003012 | AT4G16563 | 18.03 | 9.44 | 4.59 | 1.69 |
| psu.G.00003047 | AT5G23960 | 0.2 | 0.97 | 2.91 | 6.64 |
| psu.G.00003065 | AT4G28080 | 29.73 | 58.27 | 67.85 | 74.84 |
| psu.G.00003356 | AT1G15170 | 10.9 | 9.66 | 2.8 | 1.07 |
| psu.G.00003413 | AT2G44480 | 499.78 | 396.4 | 1459.07 | 1049.95 |
| psu.G.00003428 | ATMG00520 | 3.02 | 6.81 | 7.7 | 10.01 |
| psu.G.00003430 | AT1G31360 | 10.07 | 8.11 | 4.9 | 3.98 |
| psu.G.00003633 | AT1G11580 | 455.68 | 628.25 | 1065.83 | 1124.04 |
| psu.G.00003922 | AT3G51630 | 39.84 | 25.08 | 1.43 | 11.06 |
| psu.G.00004399 | AT2G02148 | 26.68 | 15.22 | 10.78 | 7.65 |
| psu.G.00004787 | AT3G58120 | 12.9 | 21.46 | 48.37 | 65.88 |
| psu.G.00004830 | AT2G27830 | 225.66 | 123.74 | 65.96 | 102.72 |
| psu.G.00004880 | No Hit Found | 26.56 | 18.04 | 11.96 | 10.35 |

| | | | | | |
|---|---|---|---|---|---|
| psu.G.00004892 | AT1G53920 | 2.79 | 6.96 | 13.72 | 21.12 |
| psu.G.00005049 | AT1G55740 | 206.74 | 141.97 | 73.51 | 65.37 |
| psu.G.00005223 | AT1G15460 | 0.93 | 2.16 | 3.94 | 6.22 |
| psu.G.00005237 | AT1G29500 | 4.01 | 3.94 | 32.35 | 27.63 |
| psu.G.00005247 | AT1G22770 | 97.03 | 67.44 | 35.97 | 46.02 |
| psu.G.00005260 | AT1G32860 | 204.12 | 190.7 | 93.75 | 92.78 |
| psu.G.00005290 | AT1G20950 | 19.35 | 7.61 | 4.27 | 3.87 |
| psu.G.00005384 | AT5G02190 | 12.05 | 20.95 | 65.45 | 37.01 |
| psu.G.00005553 | AT4G12730 | 15.81 | 31.39 | 63.2 | 67.28 |
| psu.G.00005603 | AT1G29270 | 29.66 | 25.26 | 9.2 | 1.71 |
| psu.G.00005669 | AT3G50845 | 41.25 | 23.25 | 9.48 | 14.73 |
| psu.G.00005733 | AT1G72210 | 8.68 | 8.35 | 1.32 | 2.78 |
| psu.G.00005769 | No Hit Found | 24.81 | 23.91 | 4.83 | 1.7 |
| psu.G.00006058 | No Hit Found | 424.75 | 624.14 | 3133.72 | 1749.44 |
| psu.G.00006277 | AT2G15730 | 32.63 | 27.17 | 14.18 | 14.51 |
| psu.G.00006280 | AT3G30180 | 24.68 | 27.11 | 7.18 | 8.27 |
| psu.G.00006469 | AT1G22400 | 174.21 | 178.92 | 361.93 | 591.73 |
| psu.G.00006557 | AT5G53900 | 48.92 | 35.25 | 16.53 | 22.74 |
| psu.G.00006629 | AT3G15810 | 129 | 111.97 | 46.01 | 50.69 |
| psu.G.00006702 | AT4G37870 | 484.43 | 451.85 | 129.18 | 100.81 |
| psu.G.00006918 | AT1G21410 | 17.97 | 18.61 | 7.25 | 6.31 |
| psu.G.00007035 | AT3G25830 | 9.05 | 16.86 | 29.36 | 64.14 |
| psu.G.00007228 | AT1G23380 | 12.61 | 11.65 | 3.42 | 2.84 |
| psu.G.00007293 | AT1G29460 | 1.42 | 3 | 15.64 | 21.52 |
| psu.G.00007322 | AT1G64380 | 11.82 | 13.46 | 25.86 | 45.77 |
| psu.G.00007387 | AT5G45910 | 94.92 | 85.96 | 192.77 | 195.99 |
| psu.G.00007389 | AT1G53600 | 7.42 | 4.24 | 2.99 | 2.68 |
| psu.G.00007441 | AT1G13280 | 143.63 | 177 | 431.17 | 401.91 |
| psu.G.00007925 | AT1G78100 | 17.53 | 9.03 | 8.59 | 8.64 |
| psu.G.00007998 | AT3G13080 | 5.06 | 4.6 | 2.18 | 2.47 |
| psu.G.00008023 | AT3G62340 | 16.06 | 6.68 | 3.24 | 3.41 |
| psu.G.00008126 | AT2G45580 | 28.16 | 56.73 | 11.55 | 11.85 |
| psu.G.00008212 | AT3G53980 | 86.91 | 115.37 | 42.81 | 12.11 |
| psu.G.00008253 | AT1G48790 | 25.82 | 23.18 | 10.84 | 10.6 |
| psu.G.00008362 | AT4G11650 | 0 | 1.03 | 5.45 | 13.27 |
| psu.G.00008395 | AT4G18260 | 28.13 | 20.46 | 10.12 | 9.66 |
| psu.G.00008474 | No Hit Found | 76.65 | 68.66 | 21.2 | 0 |
| psu.G.00008482 | AT4G37160 | 1.52 | 2.58 | 5.53 | 6.08 |
| psu.G.00008628 | AT4G27450 | 12.02 | 10.78 | 1.07 | 2.54 |
| psu.G.00008662 | AT3G51060 | 21.84 | 15.41 | 8.22 | 8 |
| psu.G.00008707 | AT1G17100 | 35.13 | 72.31 | 73.98 | 125.01 |
| psu.G.00008987 | AT3G61490 | 0 | 0 | 16.78 | 32.34 |
| psu.G.00009014 | AT1G22360 | 65.51 | 36.87 | 9.83 | 12.82 |
| psu.G.00009129 | AT5G23960 | 0.2 | 0.97 | 2.91 | 6.64 |

| | | | | | |
|---|---|---|---|---|---|
| psu.G.00009548 | AT2G31240 | 20.73 | 18.97 | 7.7 | 9.04 |
| psu.G.00009550 | AT1G69560 | 31.84 | 39.81 | 11.98 | 12.82 |
| psu.G.00009627 | AT2G40610 | 40.16 | 63.02 | 167.35 | 457.23 |
| psu.G.00009823 | AT4G31980 | 39.9 | 24.8 | 11.76 | 0.67 |
| psu.G.00009887 | AT2G18630 | 369.99 | 354.8 | 73.35 | 77.32 |
| psu.G.00009935 | AT5G13930 | 87.1 | 124.65 | 382.36 | 728.33 |
| psu.G.00010206 | AT3G16490 | 25.59 | 19.8 | 10.2 | 9.22 |
| psu.G.00010210 | AT2G39540 | 248.91 | 226.74 | 663.45 | 788.24 |
| psu.G.00010216 | AT3G09270 | 728.35 | 447.54 | 291.93 | 45.41 |
| psu.G.00010279 | AT4G31730 | 45.33 | 32.32 | 20.98 | 19.66 |
| psu.G.00010584 | AT2G45850 | 34.9 | 25.77 | 16.65 | 15.71 |
| psu.G.00010645 | AT5G25610 | 180.79 | 199.16 | 481.54 | 743.73 |
| psu.G.00010727 | AT1G69440 | 14.93 | 11.17 | 4.54 | 3.62 |
| psu.G.00011100 | AT2G34460 | 31.16 | 32.46 | 71.35 | 71.82 |
| psu.G.00011112 | AT5G25610 | 233.35 | 234.74 | 574.98 | 760.03 |
| psu.G.00011212 | AT1G61680 | 3.22 | 3.89 | 16 | 17.54 |
| psu.G.00011224 | AT2G20340 | 5.25 | 4.17 | 2.03 | 1.82 |
| psu.G.00011225 | AT2G20340 | 7.83 | 8.39 | 2.8 | 2.68 |
| psu.G.00011739 | AT5G62350 | 135.43 | 182.72 | 321.13 | 304.32 |
| psu.G.00011785 | AT3G22550 | 52.17 | 41.97 | 25.59 | 25.29 |
| psu.G.00011811 | AT2G18630 | 361.73 | 335.67 | 862.08 | 733.2 |
| psu.G.00011886 | AT4G34950 | 30.74 | 49.83 | 62.51 | 136.83 |
| psu.G.00012072 | AT1G59960 | 18.87 | 24.21 | 39.08 | 53.6 |
| psu.G.00012074 | AT1G29930 | 4315.64 | 7126.03 | 9192.03 | 9657.2 |
| psu.G.00012179 | AT3G53980 | 28.04 | 39.44 | 3.82 | 2.42 |
| psu.G.00012370 | No Hit Found | 20.93 | 61.41 | 95.86 | 152.16 |
| psu.G.00012434 | AT5G05960 | 164.44 | 113.93 | 72.61 | 40.38 |
| psu.G.00012568 | AT5G65690 | 132.89 | 111.42 | 46.72 | 39.39 |
| psu.G.00012572 | AT4G37870 | 223.96 | 186.12 | 93.79 | 92.96 |
| psu.G.00012585 | AT4G31950 | 17.25 | 23.76 | 59.19 | 948.39 |
| psu.G.00012593 | AT3G08570 | 69.56 | 54.07 | 31.28 | 32 |
| psu.G.00012870 | AT5G06570 | 7.64 | 7.55 | 1.84 | 0.64 |
| psu.G.00013028 | AT3G55550 | 12.88 | 22.2 | 33.37 | 33.42 |
| psu.G.00013127 | AT1G64910 | 129.45 | 136.46 | 463.83 | 541.11 |
| psu.G.00013384 | AT5G06530 | 142.44 | 174.92 | 346.71 | 371.05 |
| psu.G.00013581 | AT5G13170 | 19.16 | 16.03 | 8.05 | 1.13 |
| psu.G.00013856 | AT5G54510 | 354.31 | 229.14 | 100.69 | 109.77 |
| psu.G.00013860 | AT1G06750 | 97.18 | 75.41 | 48.46 | 18.34 |
| psu.G.00013880 | AT5G10270 | 5.9 | 4 | 2.77 | 2.65 |
| psu.G.00013979 | AT5G66940 | 32.45 | 34.31 | 13.36 | 9.54 |
| psu.G.00014288 | AT2G20340 | 9.15 | 9.04 | 2.76 | 1.47 |
| psu.G.00014323 | AT5G13930 | 5.5 | 6.38 | 13.86 | 14.15 |
| psu.G.00014576 | AT1G51850 | 7.51 | 5.24 | 3.2 | 3.55 |
| psu.G.00014631 | AT1G48410 | 17.12 | 15.63 | 7.24 | 5.6 |

| | | | | | |
|---|---|---|---|---|---|
| psu.G.00014775 | AT4G34220 | 134.38 | 135.81 | 45.9 | 38.11 |
| psu.G.00014828 | AT5G42820 | 82 | 49.4 | 39.46 | 33.76 |
| psu.G.00014947 | AT3G19000 | 80.45 | 47.19 | 31.87 | 20.2 |
| psu.G.00015090 | AT5G38280 | 6.45 | 1.8 | 1.23 | 0.43 |
| psu.G.00015158 | AT3G52950 | 128.83 | 99.4 | 57.87 | 62.95 |
| psu.G.00015248 | AT2G24050 | 22.69 | 14.77 | 10.89 | 5.6 |
| psu.G.00015265 | AT2G40116 | 8.35 | 6.9 | 2.17 | 2.49 |
| psu.G.00015569 | AT2G05760 | 8.05 | 5.45 | 2.72 | 3.33 |
| psu.G.00015723 | AT4G00210 | 26.33 | 13.52 | 4.87 | 2.37 |
| psu.G.00015871 | AT2G38110 | 10.63 | 21.11 | 77.07 | 114.92 |
| psu.G.00015965 | AT2G47000 | 3.4 | 2.12 | 1.29 | 0.64 |
| psu.G.00016004 | AT4G23530 | 21.83 | 22.76 | 9.93 | 10.07 |
| psu.G.00016018 | AT5G53950 | 29.32 | 22.15 | 11.07 | 5.72 |
| psu.G.00016158 | AT3G29320 | 166.08 | 165.05 | 72.13 | 81.42 |
| psu.G.00016160 | AT2G41890 | 23.76 | 31.64 | 8.33 | 5.85 |
| psu.G.00016225 | AT1G76130 | 37.61 | 43.86 | 17.59 | 12.85 |
| psu.G.00016256 | AT4G22010 | 61.45 | 103.81 | 165.29 | 123.07 |
| psu.G.00016341 | AT4G08150 | 29.41 | 29.89 | 7.19 | 7.74 |
| psu.G.00016375 | AT3G18640 | 9.93 | 8.03 | 4.49 | 1.2 |
| psu.G.00016434 | AT4G14230 | 58.69 | 33.18 | 26.97 | 25.3 |
| psu.G.00016671 | AT4G14740 | 132.43 | 106.61 | 65.03 | 55.37 |
| psu.G.00016791 | No Hit Found | 15.28 | 8.52 | 3.71 | 4.36 |
| psu.G.00017019 | AT5G02370 | 14.9 | 11.07 | 5.94 | 5.83 |
| psu.G.00017163 | AT4G13350 | 9.49 | 6.54 | 2.87 | 4.6 |
| psu.G.00017387 | AT1G67980 | 53.19 | 54.02 | 165.63 | 231.5 |
| psu.G.00017509 | AT5G59800 | 10.65 | 4.23 | 0.88 | 2.47 |
| psu.G.00017554 | AT5G44160 | 20.06 | 18.44 | 7.34 | 8.98 |
| psu.G.00017792 | AT4G27290 | 7.66 | 7.19 | 3.32 | 2.56 |
| psu.G.00018115 | AT4G03000 | 5.65 | 5.96 | 2.31 | 2.16 |
| psu.G.00018261 | AT4G15920 | 27.75 | 33.24 | 64.26 | 60.8 |
| psu.G.00018368 | AT3G45290 | 7.07 | 9.14 | 23.69 | 30.18 |
| psu.G.00018387 | AT4G24000 | 4.74 | 1.11 | 0.27 | 0.43 |
| psu.G.00018421 | AT1G80490 | 11.56 | 8.27 | 5.78 | 3.24 |
| psu.G.00018511 | AT1G20610 | 19.05 | 14.47 | 8.26 | 7.31 |
| psu.G.00018623 | No Hit Found | 132.65 | 71.9 | 20.47 | 11.79 |
| psu.G.00018665 | AT1G29670 | 28.67 | 14.88 | 78.85 | 105.53 |
| psu.G.00018762 | AT5G01660 | 34.67 | 28.83 | 12.83 | 16.63 |
| psu.G.00018764 | AT1G15740 | 54.44 | 33.85 | 12.95 | 22.6 |
| psu.G.00018907 | AT3G15353 | 64.63 | 91.39 | 21.73 | 29.74 |
| psu.G.00019041 | AT1G14040 | 8.54 | 8.2 | 20.35 | 17.25 |
| psu.G.00019111 | AT5G12250 | 208.2 | 364.84 | 627.65 | 602.87 |
| psu.G.00019116 | AT5G54010 | 71.02 | 77.28 | 246.4 | 323.66 |
| psu.G.00019588 | AT2G26640 | 54.43 | 47.81 | 140.39 | 152.44 |
| psu.G.00019736 | AT5G22920 | 13.71 | 10.72 | 3.06 | 2.58 |

| | | | | | |
|---|---|---|---|---|---|
| psu.G.00019935 | AT4G21820 | 15.03 | 11.38 | 7.28 | 5.92 |
| psu.G.00020154 | AT3G62820 | 18.49 | 34.93 | 42.39 | 64.64 |
| psu.G.00020175 | AT1G19715 | 18.76 | 15.18 | 6.52 | 8.21 |
| psu.G.00020275 | AT1G69530 | 1071.49 | 1228.37 | 2751.65 | 3852.96 |
| psu.G.00020283 | AT3G48990 | 73.19 | 110.48 | 173.58 | 151.47 |
| psu.G.00020330 | AT2G16390 | 4.09 | 2.64 | 1.5 | 1.27 |
| psu.G.00020384 | AT5G64200 | 91.69 | 47.04 | 41.31 | 39.74 |
| psu.G.00020460 | AT2G18500 | 23.76 | 15.94 | 8.5 | 3.69 |
| psu.G.00020858 | AT3G28345 | 8.25 | 5.72 | 4.11 | 2.78 |
| psu.G.00020913 | AT1G67830 | 117.6 | 72.12 | 55.49 | 51.12 |
| psu.G.00020934 | AT5G13630 | 82.47 | 156.23 | 176.14 | 241.98 |
| psu.G.00020981 | AT2G26640 | 13.2 | 10.16 | 42.76 | 41.4 |
| psu.G.00021029 | AT4G27450 | 271.05 | 143.52 | 60.08 | 104.45 |
| psu.G.00021080 | No Hit Found | 44.5 | 28.06 | 19.74 | 19.78 |
| psu.G.00021156 | AT4G24970 | 29.16 | 14.52 | 11.9 | 7.21 |
| psu.G.00021373 | AT3G11410 | 22.61 | 33.19 | 54.7 | 104.87 |
| psu.G.00021510 | AT3G49500 | 1.78 | 1.37 | 0.51 | 0.21 |
| psu.G.00021920 | AT1G52940 | 10.57 | 4.5 | 3.83 | 3.45 |
| psu.G.00022168 | AT3G16370 | 360.05 | 305.91 | 1020.38 | 1070.1 |
| psu.G.00022374 | AT2G04780 | 116.48 | 163.2 | 277.87 | 410.25 |
| psu.G.00022451 | AT5G62350 | 2.93 | 10.33 | 14.31 | 19.31 |
| psu.G.00022460 | AT1G76160 | 6.53 | 11.34 | 25.69 | 31.74 |
| psu.G.00022518 | AT2G03090 | 1.77 | 3.01 | 13.42 | 13.52 |
| psu.G.00022585 | AT3G07610 | 19.09 | 18.29 | 9.52 | 8.16 |
| psu.G.00022865 | AT2G20340 | 68.98 | 47.81 | 30.74 | 9.12 |
| psu.G.00022873 | AT5G22120 | 28.03 | 15.02 | 9.51 | 3.33 |
| psu.G.00023079 | AT1G77120 | 14.79 | 7.76 | 5.87 | 2.47 |
| psu.G.00023297 | AT1G43190 | 40.57 | 32.91 | 17.78 | 19.65 |
| psu.G.00023400 | AT4G35590 | 70.47 | 42.61 | 28.45 | 32.72 |
| psu.G.00023481 | No Hit Found | 120.7 | 79.14 | 34.57 | 37.24 |
| psu.G.00023693 | AT1G27210 | 31.75 | 26.63 | 14.92 | 15.31 |
| psu.G.00023853 | AT3G08570 | 69.56 | 54.07 | 31.28 | 32 |
| psu.G.00023874 | AT3G26760 | 12.86 | 21.88 | 28.45 | 31.77 |
| psu.G.00024327 | AT1G29930 | 4125.79 | 6562.41 | 9027.91 | 9155.52 |
| psu.G.00024484 | AT1G47960 | 146.37 | 79.32 | 22.9 | 9.96 |
| psu.G.00024520 | AT4G13710 | 29.77 | 54.37 | 123.81 | 135.67 |
| psu.G.00024557 | AT3G51420 | 46.85 | 26.03 | 17.58 | 22.89 |
| psu.G.00024815 | AT4G34131 | 5.62 | 11.78 | 12.56 | 13.55 |
| psu.G.00025062 | AT3G27550 | 17.3 | 11.15 | 8.06 | 8.59 |
| psu.G.00025185 | AT3G02340 | 30.47 | 13.58 | 13.05 | 8.69 |
| psu.G.00025280 | AT5G45380 | 6.37 | 2.66 | 1.67 | 1.88 |
| psu.G.00025555 | AT1G52340 | 20.65 | 19.25 | 69.36 | 51.92 |
| psu.G.00025670 | AT2G25930 | 32.06 | 20.67 | 11.73 | 14.76 |
| psu.G.00025715 | AT4G24210 | 360.5 | 294.23 | 133.14 | 136.97 |

| | | | | | |
|---|---|---|---|---|---|
| psu.G.00026202 | No Hit Found | 84.66 | 144.42 | 428.05 | 723.21 |
| psu.G.00026203 | No Hit Found | 0 | 0.66 | 10.84 | 156.22 |
| psu.G.00026236 | AT1G61680 | 1.43 | 3.04 | 11.5 | 9.62 |
| psu.G.00026413 | AT3G50700 | 8.65 | 7.05 | 2.84 | 3.14 |
| psu.G.00026484 | AT1G62360 | 13.22 | 15.52 | 2.01 | 3.82 |
| psu.G.00026597 | AT3G10950 | 30.37 | 32.32 | 12.75 | 11.41 |
| psu.G.00026877 | AT2G27610 | 7.46 | 4.06 | 2.33 | 3.31 |
| psu.G.00027187 | AT5G27670 | 404.71 | 324.23 | 199.96 | 184.63 |
| psu.G.00027253 | AT3G02100 | 26.12 | 20.92 | 11.78 | 11.06 |
| psu.G.00027279 | AT5G09280 | 5.71 | 7.2 | 20.22 | 21.1 |
| psu.G.00027282 | AT4G00490 | 0 | 0 | 3.89 | 5.26 |
| psu.G.00027382 | AT1G61680 | 1.21 | 4.12 | 9.02 | 9.28 |
| psu.G.00027558 | AT1G75180 | 17.16 | 12.52 | 7.25 | 2.47 |
| psu.G.00027565 | AT3G26310 | 2.79 | 0.46 | 0 | 0 |
| psu.G.00027640 | AT3G26330 | 66.8 | 46.79 | 29.53 | 15.03 |
| psu.G.00027768 | AT4G11650 | 61.07 | 36.21 | 21.19 | 3.21 |
| psu.G.00027887 | AT4G31940 | 2.41 | 3.61 | 8.32 | 114.05 |
| psu.G.00028016 | AT3G08900 | 676.92 | 985.38 | 1622.9 | 1772.52 |
| psu.G.00028138 | AT5G09970 | 33.89 | 29.06 | 12.41 | 2.71 |
| psu.G.00028145 | AT1G10680 | 33.45 | 35.75 | 82.14 | 79.69 |
| psu.G.00028348 | AT3G26560 | 10.52 | 8.55 | 3.5 | 2.97 |
| psu.G.00028366 | AT5G38450 | 12.69 | 19.37 | 4.69 | 1.47 |
| psu.G.00028435 | AT3G56400 | 128.13 | 75.12 | 53.64 | 30.23 |
| psu.G.00028489 | AT1G71695 | 302.3 | 170.02 | 94.44 | 142.89 |
| psu.G.00028571 | AT1G62360 | 25.63 | 25.68 | 8.58 | 9.1 |
| psu.G.00028705 | AT5G44360 | 3.21 | 4.96 | 11.84 | 8.13 |
| psu.G.00028758 | AT5G48940 | 8.76 | 5.1 | 2.76 | 1.92 |
| psu.G.00028867 | AT5G40390 | 4.82 | 7.47 | 11.08 | 21.12 |
| psu.G.00029008 | AT4G13930 | 628.39 | 872.62 | 1451.53 | 1546.69 |
| psu.G.00029042 | AT1G76420 | 18.33 | 12.02 | 6.71 | 1.31 |
| psu.G.00029126 | AT4G22880 | 31.24 | 33.73 | 100.35 | 77.11 |
| psu.G.00029132 | AT3G57520 | 1051.46 | 989.18 | 461.11 | 435.59 |
| psu.G.00029158 | AT5G23960 | 0.17 | 1.28 | 9.81 | 30.51 |
| psu.G.00029205 | AT2G18500 | 23.76 | 15.94 | 8.5 | 3.69 |
| psu.G.00029387 | AT2G24762 | 61.93 | 56.29 | 160.93 | 178.9 |
| psu.G.00029408 | AT2G38820 | 9.02 | 15.7 | 0.86 | 0 |
| psu.G.00029474 | AT4G24750 | 0 | 0 | 9.32 | 14.09 |
| psu.G.00029509 | AT3G01516 | 50.74 | 57.91 | 20.89 | 25.3 |
| psu.G.00029549 | AT3G63010 | 25.89 | 22.8 | 5.88 | 6.79 |
| psu.G.00029682 | AT5G57250 | 6.78 | 4.48 | 2.42 | 2.79 |
| psu.G.00030046 | AT2G32010 | 19.49 | 11.46 | 8.5 | 9.55 |
| psu.G.00030118 | AT5G56040 | 8.84 | 15.24 | 17.8 | 18.52 |
| psu.G.00030270 | AT5G59320 | 3262.24 | 4147.55 | 7015.24 | 7786.08 |
| psu.G.00030414 | AT5G50160 | 2.14 | 3.81 | 5.65 | 5.91 |

| | | | | | |
|---|---|---|---|---|---|
| psu.G.00030525 | AT2G21140 | 2794.4 | 3239.45 | 9388.47 | 5988.75 |
| psu.G.00030708 | No Hit Found | 6.02 | 14.39 | 37.29 | 53.99 |
| psu.G.00030733 | AT5G03760 | 4.15 | 8.83 | 28.7 | 29.43 |
| psu.G.00030773 | AT2G45550 | 28.87 | 41.53 | 72.7 | 64.33 |
| psu.G.00030860 | AT5G24530 | 173.83 | 149.23 | 82.71 | 73.14 |
| psu.G.00030929 | AT1G29500 | 11.12 | 19.99 | 49.76 | 56.98 |
| psu.G.00030938 | AT3G14280 | 51.7 | 49.76 | 15.71 | 16.59 |
| psu.G.00031028 | AT4G08950 | 9.09 | 9.95 | 27.11 | 28.26 |
| psu.G.00031291 | AT3G49260 | 23.75 | 37.05 | 92.65 | 133.04 |
| psu.G.00031299 | AT5G06900 | 25.62 | 29.94 | 57.48 | 88.57 |
| psu.G.00031560 | No Hit Found | 5.3 | 2.43 | 0 | 0 |
| psu.G.00031975 | AT2G26340 | 13.31 | 2.14 | 0 | 0 |
| psu.G.00031977 | AT2G40070 | 51.59 | 48.82 | 19.07 | 18.75 |
| psu.G.00032037 | AT5G02030 | 33.58 | 27.43 | 6.19 | 4.75 |
| psu.G.00032094 | AT1G69530 | 466.4 | 639.98 | 1274.93 | 2073.8 |
| psu.G.00032281 | AT5G66600 | 44.85 | 32.6 | 15.19 | 15.72 |
| psu.G.00032598 | No Hit Found | 132.65 | 71.9 | 20.47 | 11.79 |
| psu.G.00032772 | AT1G68640 | 38.74 | 49.91 | 15.17 | 15.25 |
| psu.G.00032783 | AT4G22880 | 78.44 | 66.64 | 232.39 | 163.9 |
| psu.G.00032805 | AT1G01200 | 43.58 | 53.39 | 91.02 | 103.93 |
| psu.G.00032900 | AT1G48130 | 29.2 | 42.46 | 7.7 | 2.82 |
| psu.G.00032932 | AT3G04290 | 435.68 | 369.63 | 880.74 | 1308.86 |
| psu.G.00032949 | AT5G15120 | 18.4 | 27.62 | 4.15 | 3.36 |
| psu.G.00033018 | AT3G18280 | 1172.11 | 1461.66 | 2414.11 | 4413.08 |
| psu.G.00033445 | AT5G58600 | 34.94 | 39.25 | 79.03 | 117.63 |
| psu.G.00033448 | AT5G45380 | 14.47 | 8.72 | 4.71 | 4.57 |
| psu.G.00033600 | AT2G27080 | 53.55 | 67.08 | 107.75 | 113.16 |
| psu.G.00033617 | AT2G26150 | 25.25 | 20.82 | 10.77 | 8.96 |
| psu.G.00033646 | AT1G58340 | 2.95 | 1 | 0.24 | 0.26 |
| psu.G.00033762 | AT5G66400 | 160.13 | 161.56 | 55.33 | 53.55 |
| psu.G.00033918 | AT1G17100 | 35.13 | 72.31 | 73.98 | 125.01 |
| psu.G.00034145 | AT3G44540 | 79.97 | 102.7 | 195.61 | 172.22 |
| psu.G.00034341 | AT5G53420 | 172.15 | 106.37 | 58.56 | 73.6 |
| psu.G.00034433 | AT1G76490 | 46.12 | 30.9 | 21.04 | 17.77 |
| psu.G.00034547 | AT3G59190 | 15.24 | 8.77 | 4.62 | 6.92 |
| psu.G.00034668 | No Hit Found | 53.43 | 39.05 | 23.38 | 14.35 |
| psu.G.00034925 | AT3G08900 | 676.92 | 985.38 | 1622.9 | 1772.52 |
| psu.G.00034940 | AT5G12330 | 65.78 | 49.56 | 21.79 | 23.47 |
| psu.G.00034967 | AT1G21410 | 96.77 | 116.54 | 41.99 | 39.73 |
| psu.G.00035210 | AT2G45220 | 11.46 | 10.37 | 1.92 | 2.4 |
| psu.G.00035252 | AT4G31500 | 27.35 | 16.26 | 11.47 | 8.55 |
| psu.G.00035328 | AT1G02500 | 3.41 | 11.84 | 0 | 0 |
| psu.G.00035439 | AT2G45580 | 260.21 | 290.97 | 113.02 | 90.33 |

**Table S3.** Primers used in this study

| Gene name | Primer | Sequence (5’-3’) | Accession No. / Gene ID |
|---|---|---|---|
| **qRT-PCR** | | | |
| *PsKS* | qPsKS-F | TTTGAATCTCCCTTTGCCACC | MH546117 |
| | qPsKS-R | TGACCATTTCCCAATCCTGTAA | |
| *PsGA20ox* | qPsGA20ox-F | TGGGTGGTCTTCAAGTGTTT | MH546118.1 |
| | qPsGA20ox-R | AGCCCTGTAATGTTTCTGTG | |
| *PsGA2ox* | qGA2ox-F | CTCCAAACCCGACACTAAACA | MH546119 |
| | qGA2ox-R | GCCCAATGTTCTTGTTCCCA | |
| *PsCO* | qPsCO-F | CGCTTTCCAGTCACTCCTTTC | KF113358.1 |
| | qPsCO-R | GCTTCTTCTTCGGCATCTCC | |
| *PsFT* | qPsFT-F | CCAAGCGACCCAAACCTA | KF113360.1 |
| | qPsFT-R | CGCCAACCTGGAGTGTAA | |
| *PsAP1* | qPsAP1-F | AGAAGAAGGAAAGGGCAATC | KF113361.1 |
| | qPsAP1-R | TTCCTCCTCACTTCTGTTGG | |
| *PsSVP* | qPsSVP-F | CGATGTTGAGCAAGGAGGTT | KC847164.1 |
| | qPsSVP-R | GCTCTAAATCAGCAGCGACA | |
| *PsSOC1* | qPsSOC1-F | CCAATGTCCGAGCAAGAAAG | KR779924.1 |
| | qPsSOC1-R | CCGTGCTTCTCGCATAACAT | |
| *PsLFY* | qPsLFY-F | ATGAGAAGGAAGGAGGGGATG | psu.G.00032564 |
| | qPsLFY-R | CTTTGGCAATGGTCTGAACT | |
| *PsGID1c* | qPsGID1c-F | TGAAGAACCTCCACCAAG | MH546122 |
| | qPsGID1c-R | CCACAAGACGACGACAAA | |
| *PsGAI* | qPsGAI-F | GAGTATGCTGTCCGAGTTCA | MH550803 |
| | qPsGAI-R | CAGGAGCAAGGAACGAAT | |
| *PsMET* | qPsMET-F | GCTGCTTTGTACTCCAGGCCC | MH038046 |
| | qPsMET-R | CTGGTGGCATTCAAGAAGTCG | |
| *PsCMT* | qPsCMT-F | TTGAATCAGGGAGTGGTAATGTG | MH038044 |
| | qPsCMT-R | TTCTTCGCCGACCCTGTTG | |
| *PsDRM* | qPsDRM-F | GAGTTTAGAGTTCCCAAGCGAG | MH038045 |
| | qPsDRM-R | GCTCTCATCCTTTCCGTCATC | |
| *SnRK1* | qSnRK1-F | CGTCGTAAGATAAAGAACAT | psu.G.00026808 |
| | q SnRK1-R | TTCCTGTAACCTACCCTTT | |
| *WAK2* | qWAK2-F | TGTTGGGTAGTTTCTGGTT | psu.G.00026316 |
| | qWAK2-R | TCGCTCCCTTCATGTTCG | |
| *5Ptase7* | q5Ptase7-F | GGTAGGGATATTTCTCACGG | psu.G.00022205 |
| | q5Ptase7-R | CTGCCCACGCAAGACACT | |
| *P450* | qP450-F | GTCCATTCCACTTGACCTG | psu.G.00002595 |
| | qP450-R | TCTTCATTATCTTCGCCAC | |
| *SBH1* | qSBH1-F | GGATCAGTTCATGGAGGCT | psu.G.00000266 |
| | qSBH1-R | TGTCGTCTGCTAGAGGGTC | |

| | | | |
|---|---|---|---|
| *Actin* | qPsActin-F | GAGAGATTCCGTTGCCCAG | JN105298 |
| | qPsActin-R | TCCTTGCTCATTCTGTCTGC | |
| **McrBC-PCR** | | | |
| *PsCO* | McPsCO-F | CACCTGCCAGAATTAGCG | KF113358 |
| | McPsCO- R | TTCCAGAAACAACCAAGC | |
| *PsFT* | McPsFT-F | GCGGCGGACCTAGATAA | KF113360 |
| | McPsFT-R | TAGTCAGGGACGCAATCG | |
| *PsAP1* | McPsAP1-F | GTTGGAGTAGAATTGTACCTAGC | KF113361 |
| | McPsAP1-R | ATGCTTCAGTCACGAGGA | |
| *PsSVP* | McPsSVP-F | CAGAGTTCGTGGCTTCAG | KC847164 |
| | McPsSVP-R | AGATGACCAGATATGTCCGT | |
| *PsSOC1* | McPsSOC1-F | AAACTTTTATCCCTCCCC | KR779924 |
| | McPsSOC1-R | CCGCACCTTAGCCTTT | |
| *PsLFY* | McPsLFY-F | GTCGCTGGACTTTATCGC | psu.G.00032564 |
| | McPsLFY-R | CAAAACATTCTGCCCGTA | |
| *PsGID1c* | McPsGID1c-F | AGTTAGGCGGGCATAGG | MH546122 |
| | McPsGID1c-R | CAAAATACCCACTTCACAGC | |
| *PsGAI* | McPsGAI-F | TCGCACCTTTAGAATACCC | MH550803 |
| | McPsGAI-R | CTCGGCTTGGCTCACA | |
| **MassARRAY Primers*** | | | |
| | Mass-F | aggaagagagAAAGTTAGGGATGGTAA TTTTTGAA | |
| | Mass-R | cagtaatacgactcactatagggagaaggctATAAC CATTAAACCAAAACACAACC | |

*The underlined parts mean mass-R inserts with a T7 promoter tag and 8 bases, and MASS-R adds 10 bases to balance Tm value.
